# Effectiveness and safety of acupoint application for chronic obstructive pulmonary disease: A protocol for updated systematic review and meta-analysis

**DOI:** 10.1097/MD.0000000000025802

**Published:** 2021-05-07

**Authors:** Hao-Yang Zhang, Han Huang, Li-Jian Pang, Xiao-Dong Lv, Wei-Dong Zheng

**Affiliations:** a Graduate School, Liaoning University of Traditional Chinese Medicine; b Affiliated Hospital of Liaoning University of Traditional Chinese Medicine; c Liaoning University of Traditional Chinese Medicine, Shenyang, Liaoning, China.

**Keywords:** acupoint application, chronic obstructive pulmonary disease, meta-analysis, systematic review

## Abstract

**Background:**

Acute exacerbation is a primary cause of repeated hospitalization and death in chronic obstructive pulmonary disease (COPD) patients. Therefore, how to control the symptoms of COPD at stable stage and reduce the number of acute exacerbation is a hot spot of medical research. Acupoint application (AA) is a significant part of external treatment of traditional Chinese medicine (TCM), Previous researches have reported that AA can be applied to the treatment of COPD. Nevertheless, its effectiveness is still inconclusive. This systematic review (SR) and meta-analysis is designed to appraise its effectiveness and safety for the treatment of patients with COPD.

**Methods:**

Eight databases will be systematically retrieved from their inceptions to February 2021. Inclusion criteria are randomized control trials of AA combined with routine western medicine interventions in the treatment of COPD at stable stage. The primary outcomes we focus on comprise clinical effective rate, TCM symptom score, quality of life, dyspnea, exercise capacity, lung function, frequency of acute exacerbation, adverse events. The research screening, data extraction, and risk of bias assessment will be conducted by 2 individuals independently, and divergence will be adjudicated by a third senior investigator. The Stata 13.1 software will be used for meta-analysis. The confidence of evidence will be classified adopting grading of recommendations assessment, development and evaluation (GRADE) algorithm and methodological quality of this SR will be assessed using assessment of multiple systematic reviews-2 (AMSTAR-2) tool.

**Results:**

This SR will provide evidence-based medical proof for the treatment of COPD at stable stage by AA combined with conventional western medicine interventions. The findings of this SR will be presented at relevant conferences and submitted for peer-review publication.

**Conclusions:**

The findings of this SR will provide up-todated summary proof for evaluating the effectiveness and safety of AA for COPD.

**Registration number:**

INPLASY 202140080.

## 1 Introduction

Chronic obstructive pulmonary disease (COPD) is a common, preventable and treatable respiratory disease characterized by persistent respiratory symptoms and airflow limitation caused by airway and/or alveolar abnormalities.^[1]^ The pathological changes are mainly airway and/or alveolar abnormalities, which are usually associated with significant exposure to harmful particles or gases.^[2]^ Many host factors such as genetic susceptibility, abnormal inflammatory response, and abnormal lung development are involved in the pathogenesis.^[3]^ Serious complications may affect the performance and mortality of the disease.

COPD is 1 of the 4 major chronic diseases in the world with high morbidity, mortality and disability.^[4]^ Chronic obstructive pulmonary disease (COPD) is a common disease that seriously endangers human health and severely affects the quality of life of patients. COPD is an important cause of death, and brings heavy economic burden to patients, their families, and society.^[5]^ According to the latest World Health Organization prediction on mortality and causes of death, with the increase of smoking rate in developing countries and the aging of population in high-income countries, the prevalence of COPD will continue to rise in the next 40 years.^[6]^ It is predicted that by 2060, more than 5.4 million people will die of COPD and its related diseases every year.^[7]^ In recent years, with the increasingly serious environmental pollution and aging problem, the incidence rate of COPD is increasing, with a higher disability rate and mortality in China.^[8]^ Epidemiological studies have shown that the prevalence rate of people over 40 years old in China is 13.7%, with nearly 100 million patients, ranking the third cause of death in China. In terms of disability adjust life year, its disease burden has ranked second in China.^[9]^

The risk factors of COPD are diverse, which can be summarized as individual susceptibility factors and environmental factors. Individual factors included genetic factors, age and gender, lung growth and development, bronchial asthma and airway hyperresponsiveness, and low body mass index.^[10]^ Environmental factors mainly include smoking, fuel smoke, air pollution, occupational dust and infection, chronic bronchitis, and social economic status.^[11]^

The pathogenesis of COPD is complex and has not been fully elucidated. Inhaling tobacco smoke and other harmful particles or gases can cause airway oxidative stress, inflammatory reaction, and protease/antiproteinase imbalance, which are involved in the pathogenesis of COPD.^[12]^ In addition, autoimmune regulation mechanism, genetic risk factors, and lung development related factors may also play an important role in the occurrence and development of COPD.^[13]^ The combination of above mechanisms can lead to the formation of COPD. The main pathophysiological changes of COPD include airflow limitation, gas entrapment and abnormal gas exchange. It can be associated with mucus hypersecretion, airway epithelial cilia dysfunction, and systemic adverse effects. Severe cases may be complicated with pulmonary hypertension, chronic pulmonary heart disease, and respiratory failure.^[14]^

COPD patients often have multiple systemic complications at the same time, which are related to the severity of the disease.^[15]^ The main symptoms of COPD are chronic cough, expectoration, and dyspnea. Cough and expectoration usually appear in the early stage of the disease, and dyspnea is the main manifestation in the later stage.

Phase of COPD can be divided into acute exacerbation stage and remission stage. In acute exacerbation stage, COPD mainly occurs in the elderly and is prone to respiratory failure, which is an important reason for patients’ repeated hospitalization and death.^[15]^ Therefore, how to control symptoms in stable stage and reduce the number of acute acute exacerbation is a hot spot in medical research.^[16]^

At present, conventional treatment for stable COPD includes pharmacological and non-pharmacological interventions. Pharmacological medications mainly contain β_2_-agonists, inhaled corticosteroids, methylxanthines, and anticholinergics, which are applied to relieve and control the symptoms. Nevertheless, no clinical evidence have shown that the current drug treatment can postpone the long-term decline in lung function of patients with COPD.^[17]^ Furthermore, nonpharmacological interventions mainly include integrative care, self-management, oxygen therapy, pulmonary rehabilitation, ventilatory support, and interventional therapy. However, it should consider the risk of adverse reactions, the inconvenience, and cost of the prolonged course of the therapy.^[18]^

Acupoint application (AA) is an important part of external treatment of traditional Chinese medicine (TCM). It is a noninvasive therapy based on the theory of meridian science of TCM, which directly applies herbs to acupoints to treat diseases and it is widely used in the prevention and treatment of chronic respiratory diseases.^[19]^ AA has the characteristics of simple operation, significant curative effect, and less adverse reactions, which has been widely recognized by the society.^[20]^ In the stable period of disease, AA can stimulate the body's healthy qi, dredge the channels and collaterals, regulate qi and blood to enhance the body's antievil ability, and control the recurrence of disease.^[21]^ This idea also fully embodies the concept of “treating pre-disease” in TCM. AA in the treatment of COPD is a typical application of this concept.^[22]^

There are many effective components and targets in the herbs of AA, and the mechanism of action is more complex. The mechanism of western medicine mainly focuses on the regulation of immunity and inflammation.

1. Inflammatory cells and mediators: some studies have shown that AA can significantly reduce the expression of IL-6, IL-2, MCP-1, and other inflammatory factors in the lung of COPD rats^[23]^; others have shown that AA can alleviate the symptoms of patients with COPD, improve the quality of life, reduce the secretion of systemic inflammatory factors and the levels of IL-8 and CRP inflammatory factors.^[24]^
2. Cellular immunity and humoral immunity: some studies have shown that AA can reduce the levels of CD3 +, CD4 +, CD4 + / CD8 + in peripheral blood, and BALF of COPD rats, but increase the level of CD8 + significantly, thus promoting the recovery of balance of both. Some studies have confirmed that AA can gradually improve the IgA, IgG, IgM levels in serum of patients with COPD, reduce the content of IgE, not only enhance the body's cellular immunity and humoral immunity, but also reduce the IgE mediated hypersensitivity.^[25]^
3. NK cell activity: the expansion of NK cells and the enhancement of anti-infection immunity are directly affected by IFN-γ and TNF-α. Studies have shown that AA can significantly reduce the levels of IFN-γ and TNF-α, improve the activity of NK cells, and improve the quality of life of patients.^[26]^

A number of clinical studies have shown that AA combined with conventional western medicine interventions has a good clinical effect on COPD.^[27]^ For patients with COPD in stable stage, compared with conventional western medicine measures, AA combined with routine western medicine interventions can improve clinical efficiency, reduce clinical symptoms, improve quality of life, and reduce the number of acute exacerbations.^[28]^ For patients with AECOPD, it can improve pulmonary ventilation function, quality of life, and clinical symptoms.^[29]^ With the increasing number of clinical trails on AA in the treatment of COPD, reliable evidence is needed. Presently, although several reviews have addressed this issue, none of them have further assessed the effectiveness and safety of AA for COPD after more new randomized controlled trials have been published.^[30–33]^ Therefore, in this study, we will provide latest and updated evidence of systematic review to evaluate the effectiveness and safety of AA for COPD.

## 2 Methods and analysis

### 2.1 Objective

The purpose of this systematic review (SR) and meta-analysis is to evaluate the effectiveness and safety of AA combined with conventional western medicine interventions in the treatment of COPD in remission stage.

### 2.2 Study registration

It is registered on the International Platform of Registered Systematic Review and Meta-analysis Protocols (INPLASY no. 202140080, https://inplasy.com/). The procedure of this protocol will be conducted according to the Preferred Reporting Item for Systematic Review and Meta-analysis Protocols (PRISMA-P) guidance.

### 2.3 Inclusion and exclusion criteria

#### 2.3.1 Type of study

Only the randomized controlled trials (RCTs) of AA combined with routine western medicine interventions in the treatment of COPD in remission stage will meet the inclusion criteria of in this SR regardless of blinding, publication type or language. Quasi-RCTs, duplicated publications, narrative publications, case reports, editorials, animal researches, and pharmacological experiments will be excluded.

#### 2.3.2 Type of participants

The participants should be diagnosed with COPD in stable phase by using clearly defined or internationally recognized criteria and aged at least 18 years old. There are no restrictions on race or gender. The cases with following diseases will not meet selection criteria:

1. respiratory disease like asthma, bronchiectasia, pulmonary fibrosis, and so on;
2. severe liver, kidney, heart disease, and so on;
3. patients were in the acute exacerbation period of COPD.

#### 2.3.3 Type of interventions

Acupoint application combined with conventional western medicine therapeutic measure should be applied in the treatment group. No restrictions were applied to the herbal regimen, acupoints selected, patching time. The same conventional western medicine therapeutic measure must be used in the comparator arm.

#### 2.3.4 Type of outcome measurements

Clinical effective rate; TCM symptom score; quality of life (COPD assessment test, St. George respiratory questionnaire, chronic respiratory questionnaire, etc); dyspnea (borg scale, visual analog dyspnea scale, modified British medical research council respiratory difficulty questionnaire, etc); exercise capacity (6-minute walking test, shuttle walking test, etc); lung function (FEV1, FVC, FEV1/FVC, TLC, RV, etc); frequency of acute exacerbation; adverse effects.

### 2.4 Search strategy

We will conduct a comprehensive retrieval on the following 8 databases: PubMed, EMBASE, Cochrane Central Register of Controlled Trials, Web of Science, China National Knowledge Infrastructure, WangFang Database, Chinese Science and Technology Periodical Database, SinoMed. The retrieval time range is from their inceptions of each database to February 2021. We will also retrieve the following databases to confirm ongoing or completed clinical trails: current controlled trials, WHO clinical trials registry, Clinical Trials.gov trials registry, The Australian New Zealand Clinical Trials Registry, Centre Watch, and Chinese Clinical Trial Registry. Grey literature will also be searched to avoid omission. The language is limited to Chinese and English. In addition, we will manually search for relevant studies according to references from previously published SRs. If any, we will try to contact the correspondence author to obtain the data we need. Subject words combined with free words method will be used for retrieval. The search strategy in PubMed is as follows and it will be adjusted according to the characteristics of each database.

#### 2.4.1 Search strategy in Pubmed

((((((((((((((((COPD) OR Chronic Obstructive Pulmonary Disease) OR COAD) OR Chronic Obstructive Airway Disease) OR Chronic Obstructive Lung Disease) OR Airflow Obstruction, Chronic) OR Airflow Obstructions, Chronic) OR Chronic Airflow Obstructions) OR Chronic Airflow Obstruction) OR Respiratory Tract Diseases) OR Respiratory Tract Diseases Obstructive Lung Disease) OR Obstructive Airway Disease)) OR “Pulmonary Disease, Chronic Obstructive”[Mesh])) AND ((“Transdermal Patch”[Mesh]) OR (((((((((((((((((((((Acupoints) OR Acupoint) OR Patch,Transdermal) OR Patchs, Transdermal) OR Transdermal Patchs) OR Point Application) OR Acupoint Application) OR Acupoints Sticking) OR Acupucture Point Paste) OR Point Application Therapy In Dog Days) OR Acupoint Sticking Therapy) OR Emplastrum Therapy) OR External Application) OR Sanfu) OR Sanfu Patches) OR Sanfu Herbal Patch) OR Sanfu Stickers) OR Tianjiu Therapy) OR Treating The Winter's Disease In Summer) OR Acupoint Herbal Patching) OR Sanfu Acupoint Herbal Patching))) AND ((((((((((randomized controlled trial[Publication Type]) OR controlled clinical trial[Publication Type]) OR randomized[Title/Abstract]) OR placebo[Title/Abstract]) OR randomly[Title/Abstract]) OR drug therapy[MeSH Subheading]) OR trial[Title/Abstract]) OR groups[Title/Abstract])) NOT ((“Animals”[Mesh]) NOT ((“Humans”[Mesh]) AND “Animals”[Mesh])))

### 2.5 Studies selection

**Figure 1:**
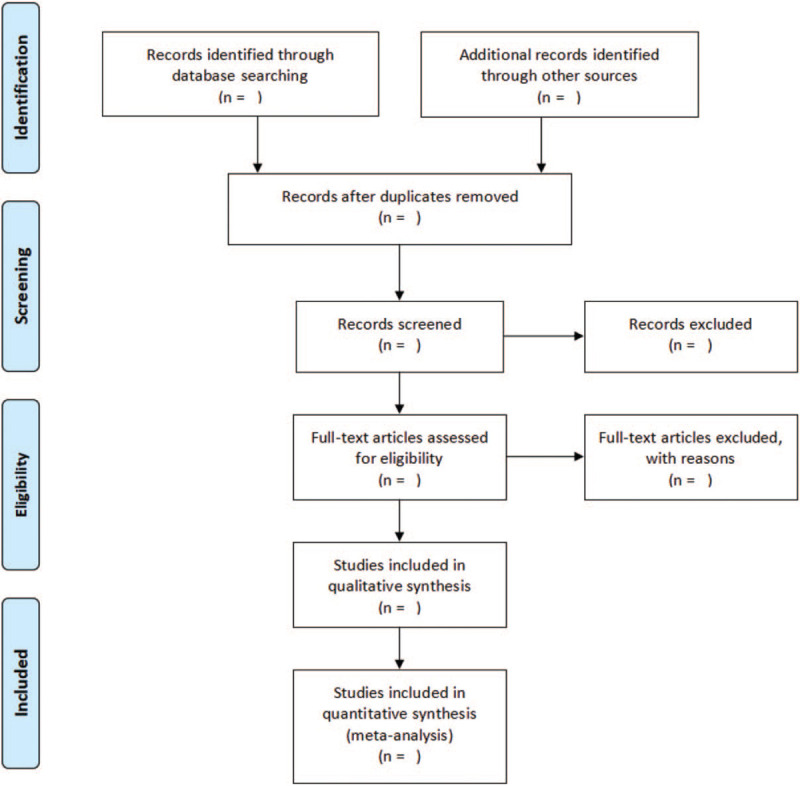
Flow diagram of study selection process legend: details of the selection process will be presented in the PRISMA flow chart.

All retrieved studies were imported into the Note Express software (version 3.0) to delete any duplicates. Two researchers (Zhang HY, Huang H) independently screened the titles and abstracts against the established inclusion and exclusion criteria and then downloaded the remaining studies for further screening by reading the full text. If any disagreements occurred, a consensus was reached through discussion or adjudication by a third senior researcher (Pang LJ). The reviewers will record all studies that do not meet the inclusion criteria and provide the rationale for their exclusion. Details of the selection process will be presented in the PRISMA flow chart (Fig. 1).

### 2.6 Data extraction

The key characteristics of the included articles were extracted independently by 2 reviewers (Zhang HY, Huang H) using a predefined form. The following data items were collected from each study: the first author, publication year, primary locality of the study, sample size (research group/control group), outcomes, range of age (research group/control group), sex distribution (male/female), diagnostic criteria, and funding. If any important information elements were missing, we attempted to contact the authors for the desired data. If any disagreements occurred during this process, the 2 reviewers reached a consensus through consultation or adjudication by a third senior investigator (Pang LJ).

### 2.7 Risk of bias assessment

We will use the Cochrane risk of bias assessment tool version 2.0 (RoB 2.0) to assess the methodological quality of RCTs. RoB 2.0 was published by the Cochrane methodology working group in 2016 and published and modified on the official website of Cochrane in 2018.^[34]^ This SR will evaluate the possibility and sources of bias in RCT from 5 areas, including risk of bias arising from the randomization process, risk of bias due to deviations from the intended interventions, missing outcome data, risk of bias in measurement of the outcome, risk of bias in selection of the reported result. Each module sets up several signal questions, and the reviewers will answer “yes,” “probability yes,” “possible no,” “no,” “not applicable” or “no information.” The overall risk of bias of RCT can be rated as low risk, high risk, and possible risk.^[35]^ Two researchers (Zhang HY, Huang H) will use ROB 2.0 to evaluate the included RCTs independently. In case of disagreement, it will be decided through discussion or by the third senior researcher (Pang LJ).

### 2.8 Dealing with missing data

In case of missing data exist in included RCT, we will contact the corresponding authors. In the event of missing data are unobtainable, intention-to-treat (ITT) analysis will be conducted, if possible, and sensitivity analysis will be performed to address the potential impact of missing data, which will be discussed if necessary.

### 2.9 Strategy for data synthesis

The Stata13.1 software (Stata-Corp LP, College Station TX77845) was used for the meta-analysis. The Q-test and *I*^*2*^ values were applied to measure the inter-study heterogeneity. When the *P* value of Q test >.1 and *I*^*2*^ < 50%, a fixed effects model was applied; otherwise, a random-effects model was used. Binary variables were expressed using the odds ratio with 95% confidence interval (CI) and continuous variables by the standardized mean difference with 95% CI. Subgroup analysis will be performed to analyze potential factors that may cause high heterogeneity. If the heterogeneity remains substantial, a narrative summary will be conducted.

### 2.10 Subgroup analysis

Where heterogeneity is significant, subgroup analysis will be implemented based on specified effect modifiers as follows: different interventions, controls, publication year, sample size, course of treatment, publication language, risk of bias.

### 2.11 Sensitivity analysis

Different levels of the risk of bias of clinical research may affect the overall effects. According to the risk of bias assessment results, we will eliminate the low-quality literature to carry out sensitivity analysis to judge the robustness of the conclusion.

### 2.12 Publication biases

If the number of included RCTs is sufficient (n ≥ 10), this SR will adopt inverted funnel plot to detect publication bias. Besides, Peters regression test will be applied for binary variables, and Egger for continuous variables to provide quantitative evidence of any publication bias.

### 2.13 Rating the confidence in estimates of the effect

Grading of Recommendations Assessment, Development and Evaluation (GRADE) algorithm will be used to assess the confidence in estimates of the effect. Five factors may downgrade the level of proof, namely inconsistency, imprecision, limitations/risk of bias, publication bias and indirectness. The overall quality of evidence may be rated as “high” “moderate” “low” or “very low.”^[36]^ The certainty of proof was independently by 2 reviewers (Zhang HY, Huang H) who had received GRADE training, and divergences in this process will be resolved through discussion or decided by a third senior reviewer (Pang LJ).

### 2.14 Assessment of methodological quality

Assessment of multiple systematic reviews-2 (AMSTAR-2) is a comprehensive critical appraisal instruments developed to measure the methodological quality of systematic review, which has been demonstrated relatively simple, reliable and effective for methodological assessment.^[37]^ It appraise all considerable steps in systematic review conduction, which contains 16 items covering topic selection, design, registration, information extraction, data statistical analysis, and discussion. The overall methodological quality can be classified as “high” “moderate,””low,” and “critically low” according to AMSTAR-2 guidance document. Online AMSTAR-2 checklist (http://www.amstar.ca/Amstar_Checklist. php) will be used to calculate and complete the scores in this study.

This process will be completed independently by 2 reviewers (Zhang HY, Huang H), and divergences in this process will be resolved through discussion or decided by a third senior reviewer (Pang LJ).

### 2.15 Ethics and dissemination

This systematic review will not require ethical approval because there are no data used in our study that are linked to individual patient data. In addition, findings will be disseminated through peer-review publications.

### 2.16 Strengths and limitations of this study

1. This study will be the latest SR to summarize the relevant evidence of AA combined with routine western medicine interventions in the treatment of COPD in remission stage.
2. This study will use the GRADE method to rate the confidence of evidence.
3. In this study, AMSTAR-2 tool will be used to evaluate the methodological quality of SR to ensure the integrity and transparency of the literature.
4. The search language is limited to Chinese and English, which may lead to omission.

## 3 Discussion

Chronic obstructive pulmonary disease has a high morbidity, mortality, and disability rate. It is a difficult problem to control recurrence in modern medicine. Acupoint application has a great benefit in the prevention and treatment of chronic recurrent respiratory diseases. Previous clinical studies have proved that AA combined with conventional western medicine treatment can reduce the number of acute exacerbations, improve symptoms and improve the quality of life in patients with stable COPD. This study will evaluate the effectiveness and safety of AA for stable COPD. Although several previous reviews have addressed this issue, a number of high-quality clinical trails on the efficacy and safety of AA for stable COPD have been published after them. This SR will provide the up-to-dated evidence on the effectiveness and safety of AA for COPD. It will provide helpful evidence for both clinical practice and future studies.

## Author contributions

**Conceptualization:** Haoyang Zhang, Xiao-Dong Lv, Han Huang, Weidong Zheng.

**Data curation:** Haoyang Zhang, Xiao-Dong Lv, Han Huang.

**Formal analysis:** Haoyang Zhang.

**Funding acquisition:** Xiao-Dong Lv.

**Investigation:** Haoyang Zhang, Xiao-Dong Lv, Han Huang, Lijian Pang, Weidong Zheng.

**Methodology:** Haoyang Zhang, Xiao-Dong Lv, Han Huang, Lijian Pang, Weidong Zheng.

**Resources:** Lijian Pang.

**Software:** Haoyang Zhang.

**Supervision:** Xiao-Dong Lv, Lijian Pang, Weidong Zheng.

**Writing – original draft:** Haoyang Zhang, Han Huang.

**Writing – review & editing:** Haoyang Zhang, Xiao-Dong Lv, Han Huang, Lijian Pang.

## References

[1] Duffy SP Criner GJ. Chronic obstructive pulmonary disease: evaluation and management. Med Clin North Am 2019;103:453–61. 30955513 10.1016/j.mcna.2018.12.005

[2] Yao Y Gu Y Yang M. The gene expression biomarkers for chronic obstructive pulmonary disease and interstitial lung disease. Front Genet 2019;10:1154. 31824564 10.3389/fgene.2019.01154 PMC6879656

[3] Liu R Wu Z Yu H. Effect of different treatments on macrophage differentiation in chronic obstructive pulmonary disease and repeated pulmonary infection. Saudi J Biol Sci 2020;27:2076–81. 32742181 10.1016/j.sjbs.2020.05.038 PMC7384370

[4] Ruvuna L Sood A. Epidemiology of chronic obstructive pulmonary disease. Clin Chest Med 2020;41:315–27. 32800187 10.1016/j.ccm.2020.05.002

[5] Rosenberg SR Kalhan R Mannino DM. Epidemiology of chronic obstructive pulmonary disease: prevalence, morbidity, mortality, and risk factors. Semin Respir Crit Care Med 2015;36:457–69. 26238634 10.1055/s-0035-1555607

[6] Diaz-Guzman E Mannino DM. Epidemiology and prevalence of chronic obstructive pulmonary disease. Clin Chest Med 2014;35:07–16. 10.1016/j.ccm.2013.10.002 24507833

[7] Varmaghani M Dehghani M Heidari E. Global prevalence of chronic obstructive pulmonary disease: systematic review and meta-analysis. East Mediterr Health J 2019;25:47–57. 30919925 10.26719/emhj.18.014

[8] Li L Zhong X Zheng A. Prevalence and risk factors of chronic obstructive pulmonary disease in kashi region, northwestern China. Int J Chron Obstruct Pulmon Dis 2021;16:655–63. 33758502 10.2147/COPD.S289620 PMC7981135

[9] Dong F Huang K Ren X. Factors associated with inpatient length of stay among hospitalised patients with chronic obstructive pulmonary disease, China, 2016-2017: a retrospective study. BMJ Open 2021;11:e040560. 10.1136/bmjopen-2020-040560 PMC7925858 33550232

[10] Hurst JR Vestbo J Anzueto A. Evaluation of COPD longitudinally to identify predictive surrogate endpoints (ECLIPSE) investigators. susceptibility to exacerbation in chronic obstructive pulmonary disease. N Engl J Med 2010;363:1128–38. 20843247 10.1056/NEJMoa0909883

[11] Huang X Mu X Deng L. The etiologic origins for chronic obstructive pulmonary disease. Int J Chron Obstruct Pulmon Dis 2019;14:1139–58. 31213794 10.2147/COPD.S203215 PMC6549659

[12] Agustí A Hogg JC. Update on the pathogenesis of chronic obstructive pulmonary disease. N Engl J Med 2019;381:1248–56. 31553836 10.1056/NEJMra1900475

[13] Brandsma CA Van den Berge M Hackett TL. Recent advances in chronic obstructive pulmonary disease pathogenesis: from disease mechanisms to precision medicine. J Pathol 2020;250:624–35. 31691283 10.1002/path.5364 PMC7216938

[14] Papandrinopoulou D Tzouda V Tsoukalas G. Lung compliance and chronic obstructive pulmonary disease. Pulm Med 2012;2012:542769. 23150821 10.1155/2012/542769 PMC3486437

[15] Cao YQ Dong LX Cao J. Pulmonary embolism in patients with acute exacerbation of chronic obstructive pulmonary disease. Chin Med J (Engl) 2018;131:1732–7. 29998894 10.4103/0366-6999.235865 PMC6048924

[16] Celli BR Wedzicha JA. Update on clinical aspects of chronic obstructive pulmonary disease. N Engl J Med 2019;381:1257–66. 31553837 10.1056/NEJMra1900500

[17] Riley CM Sciurba FC. Diagnosis and outpatient management of chronic obstructive pulmonary disease: a review. JAMA 2019;321:786–97. 30806700 10.1001/jama.2019.0131

[18] Moon JY Leitao Filho FS Shahangian K. Blood and sputum protein biomarkers for chronic obstructive pulmonary disease (COPD). Expert Rev Proteomics 2018;15:923–35. 30362838 10.1080/14789450.2018.1539670

[19] Pang LJ Liu JP Lv XD. Comparative effectiveness of 3 Traditional Chinese Medicine treatment methods for idiopathic pulmonary fibrosis: a systematic review and network meta-analysis protocol. Medicine 2019;98:e16325. 31348231 10.1097/MD.0000000000016325 PMC6709242

[20] Pang L Zhang H Lü X. Preventive and therapeutic effectiveness of Sanfu acupoint herbal patching for chronic obstructive pulmonary disease at stable stages: a systematic review and Meta-analysis. J Tradit Chin Med 2020;40:530–49. 32744021 10.19852/j.cnki.jtcm.2020.04.003

[21] Zhang HY Pang LJ Lv XD. Multiple Traditional Chinese Medicine interventions for idiopathic pulmonary fibrosis: a protocol for systematic review and meta-analysis of overview. Medicine 2020;99:e22396. 32991463 10.1097/MD.0000000000022396 PMC7523800

[22] Zhang H Liu J Liu T. Antenatal maternal medication administration in preventing respiratory distress syndrome of premature infants: a network meta-analysis. Clin Respir J 2018;12:2480–90. 30074296 10.1111/crj.12923

[23] Liu H. Acupuncture combined with acupoint application improves symptoms, daily life quality and lung function in chronic obstructive pulmonary disease patients during acute exacerbation. Zhen Ci Yan Jiu 2016;41:251–4. 29071915

[24] Zhang C Yang H Gan W. A randomized controlled trial for prevention of acute exacerbation of stable chronic obstructive pulmonary disease with acupoint application of traditional Chinese medicine: study protocol clinical trial (SPIRIT Compliant). Medicine 2020;99:e19396. 32150085 10.1097/MD.0000000000019396 PMC7478817

[25] Liu Y Zeng S Li Y. The effect of acupoint application of traditional Chinese medicine for the treatment of chronic obstructive pulmonary disease: a protocol for systematic review and meta-analysis. Medicine 2020;99:e22730. 33120771 10.1097/MD.0000000000022730 PMC7581166

[26] Wu JJ Zhang YX Xu HR. Effect of acupoint application on T lymphocyte subsets in patients with chronic obstructive pulmonary disease: a meta-analysis. Medicine 2020;99:e19537. 32311923 10.1097/MD.0000000000019537 PMC7220479

[27] Wang JM. Clinical study on Acupoint Application in the treatment of stable chronic obstructive pulmonary disease. Nei Mongol J Tradit Chin Med 2020;39:118–20.

[28] Zhang WW Zhou MY Wang ZX. Clinical observation on 56 cases of chronic obstructive pulmonary disease in stable stage treated by acupoint application of traditional Chinese medicine in winter and summer. Hunan J Tradit Chin Med 2020;36:04–6.

[29] Chang P Wang BQ. Observation on the clinical efficacy of Acupoint Application of traditional Chinese medicine in patients with stable chronic obstructive pulmonary disease. World Latest Med Inf Digest 2019;19:245–6.

[30] Lin FX Zheng JH Zhong MM. Meta analysis on prevention and treatment of chronic obstructive pulmonary disease with Sanfu plaster [J]. Guangxi Tradit Chin Med 2016;39:76–80.

[31] Wu D Yang C Tong Jb. Meta analysis of acupoint application in the prevention and treatment of chronic obstructive pulmonary disease. World Sci Technol Modern Tradit Chin Med 2019;21:2491–8.

[32] Hu HY Sun MY Zhu ZY. Systematic review and meta analysis of Acupoint Application in the treatment of stable chronic obstructive pulmonary disease. Shanghai J Acupun 2019;38:932–40.

[33] Wang Q Zhao CH. Meta analysis of randomized controlled clinical studies on acupoint application in the treatment of COPD in remission. Modern Chin Med 2014;34:44–8.

[34] Yang ZR Sun F Zhan SY. Risk of bias assessment series: (2) introduction to bias Assessment Tool 2.0 of parallel design randomized controlled trials. Chin J Epidemiol 2017;38:1285–91. 10.3760/cma.j.issn.0254-6450.2017.09.028 28910948

[35] Liu K Sun dQ Liao X. Interpretation of bias Risk Assessment Tool 2.0 in randomized controlled trials. Chin J Evid Cardiovasc Med 2019;11:284–91.

[36] Pollock A Farmer SE Brady MC. An algorithm was developed to assign GRADE levels of evidence to comparisons within systematic reviews. J Clin Epidemiol 2016;70:106–10. 26341023 10.1016/j.jclinepi.2015.08.013 PMC4742519

[37] Shea BJ Reeves BC Wells G. AMSTAR 2: a critical appraisal tool for systematic reviews that include randomised or non-randomised studies of healthcare interventions, or both. BMJ 2017;358:j4008. 28935701 10.1136/bmj.j4008 PMC5833365

